# The Yentl Syndrome: A Case Report

**DOI:** 10.31729/jnma.7004

**Published:** 2021-09-30

**Authors:** Barkadin Khan, Anjan Kumar Basnet

**Affiliations:** 1 Shahid Gangalal National Heart Center, Kathmandu, Nepal; 2 Health Foundation Nepal, Lalitpur, Nepal

**Keywords:** *cardiovascular disease*, *case report*, *women*

## Abstract

Cardiovascular disease, including ischemic heart disease, is one of the most common causes of death and disability in both sexes. The traditional concept of ischemic heart disease as a "man's disease" is debunked. Yentl syndrome is used to describe the underdiagnosis of ischemic heart disease in females and its associated effects. This article reports a 48-year-old female presented to the emergency department with acute epigastric discomfort. Her initial diagnostic tests did not reveal any abnormalities, and she was discharged. Subsequently, after four days, she again visited the emergency department with chest pain, the evaluation of which furthermore revealed no abnormalities. However, we admitted her. After 40 hours of hospitalization, her evaluation revealed anterior wall ST elevation myocardial infarction, and she underwent emergent reperfusion via coronary catheterization. This combination of atypical signs and symptoms and chances of delayed manifestations in the diagnostic investigations provides evidence for a need for thorough assessment in a female with chest pain.

## INTRODUCTION

Females represent nearly half of total deaths from cardiovascular (CVD).^1^ CVD in women; however, may go unrecognized, particularly at younger ages, as they are more likely than men to present atypical symptoms such as abdominal discomfort, fatigue, sleep disturbance, shortness of breath, weakness, indigestion, and anxiety.^2^ Endothelial dysfunction, small vessel size, and diffuse atherosclerosis are the causes of ischemia without evidence of a blockage.^3-4^ We report a female patient diagnosed with ischemic heart disease after two days of admission despite having normal investigations in the initial workup.

## CASE REPORT

The patient is a 48-year-old female with no risk factors of atherosclerosis causation presented to the emergency department (ED) of Shahid Gangalal National Heart Center with complaints of epigastric pain for the past four days. She described the pain as mid-sternal, continuous, radiating towards the left arm, and associated with sweating but not related to exertion. She could not recall the pain in the past and denied tobacco smoking, alcohol intake, or illicit drug abuse. She also does not have any family history of coronary artery disease or premature deaths.

**Figure 1:**
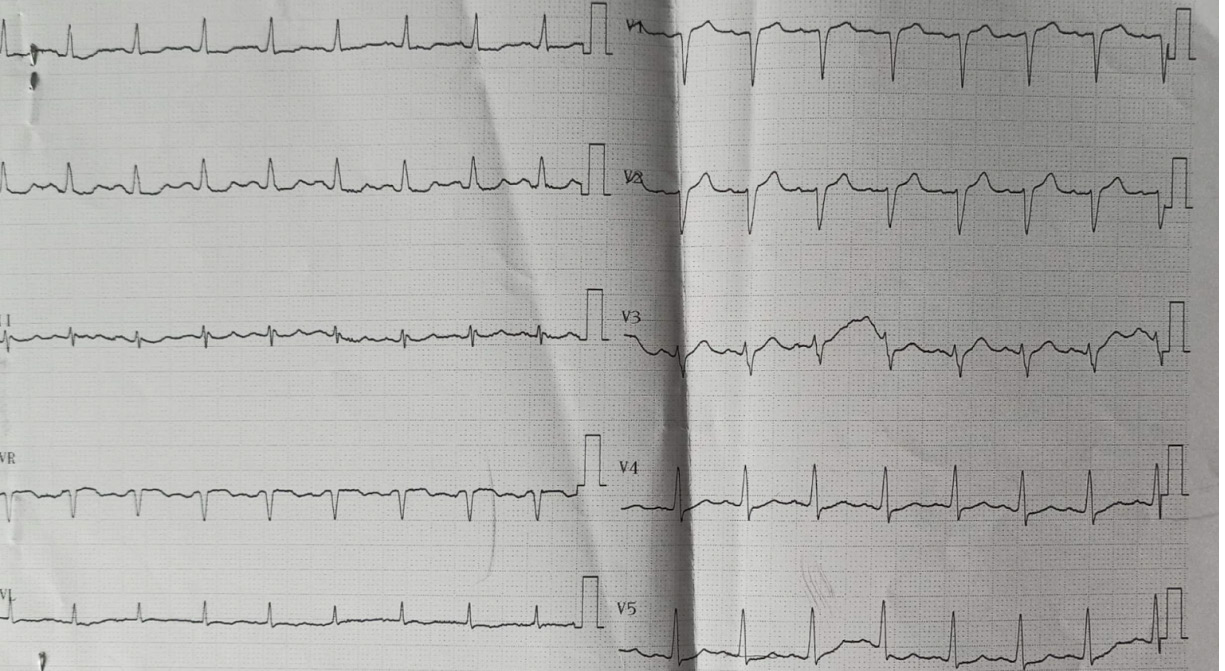
Initial electrocardiogram at the emergency department.

Upon presentation to the ED, her initial vitals were: blood pressure 170/110 mm Hg, pulse 101 beats/min, regularly regular, temperature 97.5°F, respiratory rate 20 breaths/min, and oxygen saturation 96% in the room air. Her cardiac evaluation was stable without any murmurs or rubs, and respiratory examination revealed bilaterally clear lungs with normal breath sounds. Her initial 12-lead electrocardiogram (ECG) demonstrated normal sinus rhythm with mild tachycardia (107 beats/min), normal axis, and no ST or T waves deviations (Figure 1). Her screening echocardiography and cardiac biomarkers also did not reveal any abnormalities. In ED, she was managed with intravenous antihypertensive and antacid. Her pain subsided on its own without the administration of nitrates or morphine. She was admitted to the cardiology department to evaluate her chest pain and started treatment with aspirin, statin, beta-blocker, ACE inhibitor before a planned coronary angiography after 72 hours. During the hospital stay, the etiological investigations such as fasting blood glucose level, lipid profile, renal function test, complete blood count did not reveal any risk factors for coronary artery disease.

**Figure 2:**
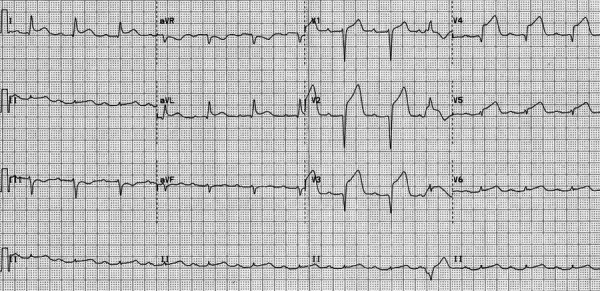
Electrocardiogram during the episode of chest pain.

**Figure 3:**
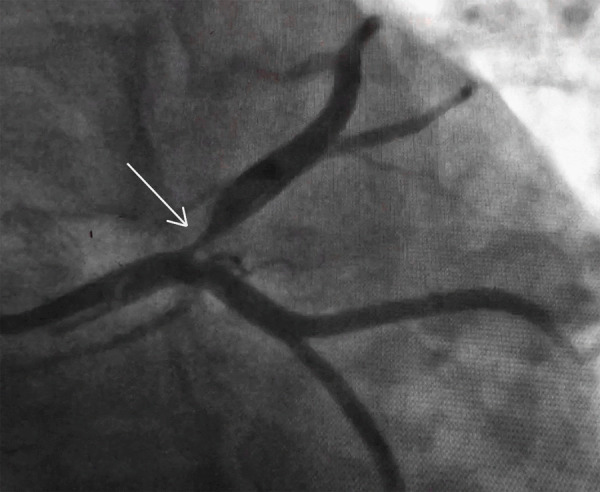
Coronary Angiography Right Anterior Oblique caudal view shows significant occlusion of ostial LAD artery.

After 40 hours of admission, she again complained of retrosternal chest pain, a severe tearing type associated with sweating and nausea. The pain lasted for 30 minutes. ECG performed revealed significant ST elevation in leads V1, V2, V3, V4, and ST depression in leads II, III, and aVF (Figure 2). Screening echocardiography showed hypokinetic left anterior descending artery territory, mild mitral regurgitation, and left ventricular ejection fraction (LVEF) of 40%. Coronary angiography performed stat revealed significant occlusion in the ostial left anterior descending (LAD) artery while the right system was normal (Figure 3).

The cardiac catheterization team was notified and mobilized for the preparation of percutaneous coronary intervention (PCI). Emergent rescue PCI with drug-Eluting stent implantation was performed via the right radial approach. Complications like slow flow or no-reflow were not observed, and thrombolysis in myocardial infarction (TIMI) 3 flow was restored. The patient was transferred to the intensive care unit (ICU) for further management, where she reported improvement in chest pain. The ECG changes improved within a few hours of the procedure. The patient was stepped down to the ward after 48 hours of ICU stay and then discharged after three days of stay in the ward.

On six months of follow-up following discharge, she expressed good exercise tolerance with no chest pain, and her vitals were within the normal range. ECG revealed no new changes, and echocardiography showed mildly hypokinetic anterior wall and improved LVEF of about 50%.

## DISCUSSION

The literature suggests that when women present like men (with 'male-pattern obstructive CHD), they are more likely to be assessed and treated like men. As characterized by the 'Yentl syndrome' depicted in the Barbra Streisand movie of the same name, Dr. Bernadine Healy described this term in 2001 to call attention to the paradox of adverse outcomes of women with ischemic heart disease (IHD), as well as the under-diagnosis and under-treatment of women.^5^ The harmful ischemic heart disease gender gap is widest in relatively young women, where MI mortality is twofold higher in women younger than 50 years compared with age-matched men.

Perhaps the most significant contributor to this is that their doctors simply miss their diagnosis. It is partly because women often don't have the "Hollywood heart attack" with chest and left-arm pains. Women, mainly young, may present without any chest pain but stomach ache, breathlessness, nausea, and fatigue. These symptoms, often described as "atypical," may lead to the under-appreciation of risk associated.^2^ Similar situation occurred with our patient, who visited ED initially for epigastric pain. As she did not have a typical presentation of Acute Coronary Syndrome (ACS) and her evaluation, such as ECG and cardiac markers, also did not reveal any abnormalities, so she was discharged with a provisional diagnosis of anxiety. But on a subsequent visit to ED for chest pain with left arm radiation, we admitted her to our cardiology department to further evaluate her chest pain, and we discovered the unexpected outcome as a left ostial LAD occlusion ST-segment myocardial infarction.

Studies have demonstrated under-treatment of women with medication, including lower rates of aspirin and ACE inhibitor use in stable women than men, lower rates of ACE inhibitor, beta-blockers, and statins in ACS women than men.^6^ ACS women undergo fewer index angiograms, percutaneous coronary interventions, and coronary artery bypass grafting (CABG) than their male counterparts.^7^ Atherosclerotic plaque in women is less fibrotic and contains more lipid-filled foam cells, owing to more significant potential for reversibility but potentially increasing vulnerability for rupture and thrombus formation.^5-6^ But women's symptom misinterpretation may lead to the wrong diagnosis and treatment with potentially unfavorable consequences. The presence of typical symptoms does not have any difference in the disease diagnosis between women and men.^8-9^ However, when prodromal symptoms are recognized early in women, they have better survival than men.^10^ This case report presents an interesting case of missing CAD initially. Heightened attention in the evidence-based approach in diagnosing and managing CHD in females is recommended.
